# Deliver Smart, Not More! Building Economically Sustainable Competitiveness on the Ground of High Agri-Food Trade Specialization in the EU

**DOI:** 10.3390/foods12020232

**Published:** 2023-01-04

**Authors:** Marius Constantin, Juan Sapena, Andreea Apetrei, Simona Roxana Pătărlăgeanu

**Affiliations:** 1 Agri-Food and Environmental Economics Department, Faculty of Agri-Food and Environmental Economics, Bucharest University of Economic Studies, 010374 Bucharest, Romania; 2 Economics, Business and Marketing Department, Catholic University of Valencia, 34 Corona Street, E-46003 Valencia, Spain

**Keywords:** competitiveness, economic sustainability, agri-food value chains, agriculture, factor endowments, productivity, trade flows, revealed comparative advantage, strategic actions

## Abstract

Competitiveness has always been a multifaceted illusive concept, which has made it a real challenge for scholars and practitioners to find the most suitable measurement tools to completely encapsulate all the complex nuances of competitiveness. This becomes even more of a challenge when approached in relation to particular economic sectors. The agri-food sector is no exception, especially when considering all its interconnections with the other sectors: water, energy, transport, waste. All of them impact the achievement of the Sustainable Development Goals (SDGs). Similarly, scholars have been debating the meaning of sustainability for decades, some even arguing that it is a political, subjective, and, in some cases, self-contradictory concept. As far as the sustainability of agricultural competitiveness is concerned, the literature is still developing. It is much more focused on fostering environmental competitiveness, and less attention was paid to the strategies designed to capitalize on sustainable economic competitiveness—a concept that has attracted divergent opinions in the literature, mainly due to ambiguity. Thus, instead of falling into the pitfall of vagueness, this paper was aimed at bringing its contribution to this field by undertaking the research objective of exploring a single facet of sustainable agricultural competitiveness: the economic facet. Hence, this paper proposes the construction of the sustainable economic competitiveness index (*SECI*) with direct application for agri-food value chains. It consists of three attributes: (a) factor endowments, resource independence; (b) agricultural chain performance; and (c) national agricultural chain strategies and policies. In this study, *SECI* was tested against the cereal chain for a selection of EU countries, based on the data taken over from FAOSTAT and INTRACEN Trade Map, in the case of the 2011–2020 period. Various statistical and econometric methods were used to test the robustness of *SECI*. Results stand as proof that building sustainable agricultural economic competitiveness relies on a mix of strategic actions. The key vector in this mix is that trade flow patterns and policies must be calibrated in accordance with national factor endowments in order to achieve high levels of *SECI*. To add more managerial implications, this paper argues for the smart delivery of agri-food products with high added value instead of focusing on exporting big volumes of raw agricultural materials with little added value.

## 1. Introduction

This study is based on the holistic perspective provided by Feurer and Chaharbaghi on competitiveness–it “is relative and not absolute” [1]. Measuring competitiveness is dependent on what this concept is perceived to represent by the person or team wanting to measure competitiveness levels. In addition, Feurer and Chaharbaghi argue for the necessity to analyze the economic sustainability of competitiveness, as one of its components. According to the same authors, the balance between factors of conflicting nature must be maintained in equilibrium with the aim of ensuring the sustainability of competitiveness. Through strategies, by “acting and reacting”, potential must remain high to ensure competitiveness, even during a changing environment [2,3].

### 1.1. A Brief Literature Review of the Concept of Competitiveness

Before tapping into the sustainability of competitiveness, it is important to revisit the traditional approach regarding competitiveness. It sheds light on the ability to actively compete in the global open market of goods and services, based on the laws of demand and supply [4]. In this regard, the price is one of the aspects that can be analyzed when quantifying the degree of competitiveness—Black et al. [5] argued that suppliers are competitive as long as their prices are at least as low as the prices of their market rivals. Building competitiveness relies on the financial capabilities of stakeholders who act and react in a global dynamic economic environment in the direction of efficiently mixing the optimal production factors that add the maximum value to a product, service or sector. Yet, competitiveness is more complicated than that—it is more than productivity growth, efficiency measurement, cost management or international engagement. In the literature, competitiveness has also been linked to the concept of economic performance [6], which, in some cases, can even be measured by comparing relative inflation rates [7]. Balkyte and Tvaronavičiene [8] classified competitiveness in six categories: (a) at firm or company level; (b) at sector level; (c) regional competitiveness (referring to territories); (d) national competitiveness (at country level); (e) bloc competitiveness; and (f) external competitiveness (referring to global/international competitiveness).

The concept of competitiveness has a long history in the scientific literature. Of course, Adam Smith laid the foundation of economics that was grounded in the power of unencumbered competition and specialization across countries. Following Adam Smith, Ricardo’s comparative advantage was explained based on price and cost differences, which was later refined by Heckscher and Ohlin [9,10]. A solid technique for measuring the level of competitiveness was provided by Balassa—through the revealed comparative advantage (RCA), which is also called the Balassa index [11]. Michael Porter is another pioneer that has added fundamental layers to the literature of competitiveness and has complemented Adam Smith’s work [12]. However, unlike Smith’s trade theory grounded on the premises that endowments are the source of wealth, Porter argued that there is another element that creates wealth besides factor endowments—that is choice [13].

Other early studies that were aimed at defining competitiveness provided valuable insight to scholars and practitioners. For example, in 1985, Scott and Lodge [14] argued that competitiveness refers to the ability of a country to: (1) create; (2) produce; and (3) distribute products/services in international trade flows with the condition of generating rising returns on its resources. Later, in 1988, Fagerberg [15] also had a macroeconomic approach and defined competitiveness as the ability of a nation to achieve its economic policy goals—but income growth and high levels of employment especially, while not encountering any difficulties as far as the balance of payments is concerned; in 1996, Waheeduzzaman and Ryans [16] reviewed the concept of competitiveness and put into the spotlight the fact that it can be approached from two different angles: (a) from the perspective of a company (firm), therefore what it is called the micro perspective; and (b) from the perspective of nations—therefore the macro perspective of competitiveness.

Competitiveness can be a source of wealth for any market economy and it does not exclude any economic sector [17]. Regarding the competitiveness of the agri-food sector, a comprehensive review paper was elaborated by Mizik [18], who presented the multidimensional facets of agricultural competitiveness, as well as the most frequently used techniques designed to quantify the level of competitiveness. In his review, Mizik highlights that nations are competitive in various manners: (a) price competition can dominate in some agri-food chains; (b) agricultural and yield productivity acts as a source of competitiveness; (c) through added-value, semi-processed and processed foods imply more competitiveness generally than in the case of raw agricultural materials (which was also demonstrated in another study of the same author [19]); (d) exporting patterns—in the sense that export-oriented countries are more likely to be resilience and maintain their competitive position in the global market. Among all these factors of influence, three are considered the most important based on reviewing the literature: (1) sophisticated agri-food products with higher added value; (2) highly efficient technology and profitable production; and (3) favorable trade policies.

Economic competitiveness in terms of international trading is also influenced by factors such as political conflicts [20,21,22]. The most recent conflict with direct impact on the trade flows with agri-food products is the Russian–Ukrainian conflict [23]. It occurred in the late February 2022, and it has caused the global disruptions in food supply chains [24], on top of the economic and supply shocks caused by the COVID-19 pandemic [25,26]. This conflict adds more pressure on the price of cereals due to the uncertainty that wheat supplies need to manage [27,28], and it also worsens global food security, as well as the implementation of the 2030 Agenda for Sustainable Development [29,30]. There are authors who believe that such a conflict will compromise the progress made over the past decade through the Sustainable Development Goals (SDGs) in the area of food security [31]. Not only does the Russian–Ukrainian conflict imply an increase in geopolitical tensions and socio-economic struggles [32,33], but it also involves a reconfiguration of economic competitiveness [34]. Achieving and maintaining a sustainable competitive advantage in terms of agri-food products trading has been difficult even before the Russian–Ukrainian conflict or the COVID-19 pandemic, due to many influencing factors, as identified in the literature: the conflicting sustainability-related goal of achieving global food security simultaneously with aiming for economic development obtained through high product specialization and levels of competitiveness [35,36], finding pathways for mitigating the impact of agricultural activities on climate change [37,38], managing trade wars [39,40], and other relevant factors.

### 1.2. A Brief Literature Review of the Concept of Sustainability

As far as sustainability is concerned, the United Nations (UN) Brundtland Commission defined sustainability as “meeting the needs of the present without compromising the ability of future generations to meet their own needs” [41]. There are many attempts in the literature at defining sustainability in general [42,43,44], which mostly converge with the UN’s vision. Furthermore, the literature also tends to converge towards the idea that sustainability has three dimensions: social, economic and environmental [45,46,47,48,49]. Regarding the agricultural sector, Tilman et al. [50] argue that this sector is among those with strategic managerial implications in terms of the global effort to foster sustainable agricultural practices. These practices are considered to meet current and future nutritional societal needs and that maximize societal net benefits, while having all costs and benefits of the practices taken into account [50]. Moreover, since the socioeconomic and environmental interconnections of the agricultural sector with other sectors (energy, manufacturing, transportation) were acknowledged [51,52,53], it is the role of politics and ethics to find pathways for the harmonization of societal and economical needs with the expected environmental impacts of the agricultural practices [54,55].

There are also scholars who argue that the meaning of sustainability cannot be clearly defined, because the set of terms used to describe it is too abstract. For example, in relation to the UN’s definition of sustainability, Ramsey [56] argues a comprehensive and accurate interpretation of the word “needs” is impossible—it can be variously interpreted, making it difficult to accept or reject what is correct or incorrect based on the multitude of interpretations of the terms used to define sustainability, such as the word “needs”. In relation to the many targets designed to measure the achievement of the SDGs, Ramsey has taken Wu and Wu’s work [57] into account and stated that the existence of “hundreds of indicators, indices and indicator initiatives” undermine the goal of a coherent and precise definition of sustainability. Moreover, Ramsey shared Jacobs’ vision [58] and argued that sustainability is a political concept that was and always will be contested in some manners, mainly because of the conflicting expectations that scholars and practitioners have in relation to it. Owen [59] stated that sustainability is “one of the least meaningful and most overused words in the English language”, while Johnston et al., emphasized that sustainability can be internally self-contradictory—an oxymoron [60].

On some level, the empirical analysis carried out in this research paper accepts the limits of defining and measuring sustainability, as conveyed by Ramsey [56]. Instead of aiming to encapsulate in a composite indicator what is generally accepted in the literature to be “sustainability”, with all its components, this study is limited to what is accepted to be economic sustainability. The study of the dynamic interactions between profitability, resources management, capabilities and competitive advantage is essential for the elaboration of a resource-based strategy that would allow for putting the proper mechanisms in place for creating a sustained competitive advantage over time [61,62,63].

### 1.3. Reviewing the Nexus of Sustainability, Competitiveness and the Economic Dimension

Regarding the link between sustainability, competitiveness and economic growth, they are interconnected. Fostering a better entrepreneurial climate, favorable for innovation and investment, boosts the growth potential, triggers competitiveness, delivers economic growth, as well as improvement of the quality of life [64,65,66]. Barbier [67] argued that building any sustainable economy should be grounded in the premises of economic development, conventionally referring to Meier’s conditions [68]: (1) the systematic increase in income per capita over longer periods of time, and (2) the avoidance of an unequal distribution of income. However, more is needed in order to achieve sustainable economic development. Improvements can become questionable without solid strategies to sustain the ecological and social aspect of improvements (besides the economic ones), as well as maximizing the positive outcomes across the whole system through a robust and comprehensive trade-offs process.

Fostering sustainable competitiveness and harnessing it in the light of the intelligent use and management of resources is one of the key aspects of any sustainable economy [69]. It also involves at least maintaining the same level of competitiveness, if not possible to improve it [70], which, in some cases, can refer to the position of a product or service in the global market [71]. Among other factors, achieving high levels of wellbeing and sustainable economic growth is dependent on intense competitiveness [72,73,74].

Ensuring high levels of sustainable competitiveness is the goal of any competitive strategy. Coyne [75] argued that there are three conditions that need to be met in order to achieve a sustainable competitive advantage: (1) the product/service marketed should be differentiated based on favorable producer attributes if comparing his/her product or service to that of the competitors; (2) no matter their nature, capability gaps need to be the direct of consequences of the product/service attribute differences mentioned at point (1); and (3) capability gaps and the difference in product/service attributes must last over longer period of time. Coyne’s perspective is convergent with Hall’s view [76], in the sense that sustainable competitiveness results from achieving powerful capability differentials. Success can be looked upon as delivering, maintaining and constantly improving all the sustainable competitive advantages one company, economic sector, bloc or nation has compared to their competitor(s) [77].

### 1.4. The Research Objective and the Novelty Factor in the Context of Literature Gaps

While some part of the literature is dedicated to the analysis of factor endowments, technological capabilities, to the role of foreign direct investment in agriculture, the role of governmental and to multinational policies on the generation and capitalizing on a country or sector’s competitive advantage [78,79,80,81,82], there is a gap in terms of studies dedicated to the exploration of the sustainability of agricultural competitiveness. This empirical study was aimed at bringing its contribution to the literature with a perspective on the economic sustainability of agricultural competitiveness, from the perspective of trade specialization, trade policies, factor endowments and agricultural chain efficiency.

Thus, the objective of this research was to construct a composite indicator that would encompass the most essential characteristics of what is generally accepted in the literature as sustainable economic competitiveness. Considering that the focus of this study was the agricultural sector, we proposed only one agri-food chain for this analysis—the cereal chain. For the construction of the sustainable economic competitiveness index (*SECI*), three main types of indicators were considered, according to the following division: (a) factor endowments and resource independence; (b) agricultural chain performance; (c) national agricultural chain strategies and policies. Hence, the economic sustainability dimension of the index is ensured by all its attributes and components. A selection of EU countries was the subject of this research, and the sustainable economic competitiveness of the cereal’s chain was analyzed based on statistical and econometric techniques.

This empirical research adds more layers to the literature with a comprehensive approach to the traditional sustainability and competitiveness paradigms. Instead of falling into the illusiveness of those two concepts, this paper proposes a rather narrow and niched path for the exploration of a single specific facet of both competitiveness and sustainability; a facet that has a common ground for both concepts, and that is the economic valence. Thus, it is argued in this research paper that agricultural sustainable economic competitiveness can be achieved through a mix of strategic actions of decision-makers, which needs to be grounded in a solid factor endowment foundation and efficient trade specialization policy. In addition, it is highlighted that building economically sustainable competitiveness does not necessarily imply focusing on production quantity, but rather on adding more value to the final product through a performant value chain management system. The novelty factor of this paper also resides in the constructed sustainable economic competitiveness index, which confirms that delivering strategically is key to becoming highly competitive in the dynamic European context.

This section is followed by methodological explanations—data selection, extraction, and processing techniques—as well as by the description of the statistical and econometric instruments utilized to construct and test the composite indicator. The next section, Section 3, was dedicated to research results and discussion. The final section, Section 4, touches upon the conclusions of this empirical study. It is highly focused on managerial implications, current research limitations and directions for future research avenues.

## 2. Materials and Methods

Defining or exploring the concept of competitiveness can be redundant unless a measurement system is used to determine the level of competitiveness of a product, service, company or sector from certain perspectives [83]. Of course, the literature consists of papers dedicated to encompassing as much as possible of the characteristics of agricultural competitiveness. In this regard, Notta and Vlachvei [84] constructed a composite index for measuring the level of competitiveness of EU food and beverage manufacturing industries; Singh and Sain [85] formulated a composite index designed to express the competitiveness of India’s agricultural export. Fischer and Schornberg [86] proposed an industrial competitiveness index as a measurement technique for the economic performance of EU food and drink manufacturing industries based on the profitability, productivity and output growth factors. Besides the body of literature consisting of composite indices designed to measure the level of agricultural competitiveness, the literature also consists of papers dealing with the measurement of agricultural competitiveness through the lens of Porter’s Diamond model [87,88,89], which focuses on at least four attributes, as described by Michael Porter [90]: (a) firm strategy, structure and rivalry; (b) factor conditions; (c) related and supporting industries; and (d) demand conditions. Besides those four attributes, Porter also acknowledged the influence of governmental forces, as well as luck—both considered impactful in terms of fostering national competitive advantage. Of course, Porter’s framework can be further refined, since competitiveness is such a complex concept. For example, Bosch and Prooijen [91] brought into the spotlight of competitiveness research that Porter had initially missed an essential element of analysis, which is national culture.

However, there is no global composite scale to quantify the level of agricultural competitiveness in all its dimensions. The purpose of this paper was not to measure competitiveness in light of the characteristics that define it entirely, but rather focus on quantifying the agricultural competitiveness and its economic sustainability specifically. The environmental component of the sustainability of competitiveness is outside the scope of this article, but can be the object of further research in this field, especially if considering the delicate relationship between the agricultural sector and climate change [92,93,94,95,96].

**Figure 1:**
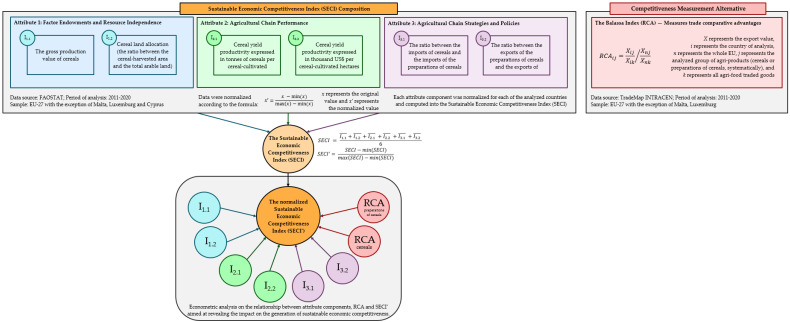
Research diagram flow.

Thus, in line with the research objective, an index was constructed with the aim of measuring the level of sustainable economic competitiveness—the sustainable economic competitiveness index (*SECI*). Three attributes were considered when developing this index: (a) factor endowments, resource independence; (b) agricultural chain performance; and (c) national agricultural chain strategies and policies. As described in Table 1, each attribute has two components (indicators), and their selection was grounded in the literature—as per the reasoning column. After data were extracted from the relevant databases, the next step in the construction of the composite index was to normalize data. Carrying out this procedure (further detailed in Figure 1) ensured the same comparison ground for each observation within the sample in relation with each index component.

Regarding the sample, this study was dedicated to the EU area with the purpose of identifying solutions for the improvement of the Common Agricultural Policy (CAP) in terms of harnessing sustainable economic competitiveness through the lens of *SECI*. The CAP is one of EU’s main policies; a driver of change in the European agriculture, market and rural areas; a policy with significant economic impacts internationally [97,98,99,100]. With its 60th anniversary in 2022, the CAP is financed by: (a) the European agricultural guarantee fund (also referred to as “the first CAP pillar”), which is designed for income support schemes; and (b) the European agricultural fund for rural development (also referred to as “the second CAP pillar”), which is designed to ensure the financial framework of proper rural development [101]. In 2021, the CAP expenditure in the total EU expenditure was close to 25% [102]. The importance of this policy for the EU is implicit, considering the numerous CAP dimensions: economic, societal, environmental and food security [103,104,105]. Thus, selecting the EU countries as the sample for this study sets this research in a framework designed to generate practical solutions for delivering more efficient CAP measures in terms of capitalizing on sustainable economic competitiveness.

In this study, two types of agri-food products were analyzed: (1) cereals; and (2) preparations of cereals. This selection is optimal for highlighting the need for a coherent CAP policy that is able to ensure the smart delivery of agri-food products with high added value instead of following the patterns of exporting big volumes of raw agricultural materials with little added value. To support this approach, the literature consists of similar papers dedicated to the study of relative trade advantages (as a form of economic competitiveness) for bulk primary raw agricultural commodities, such as cereals, in comparison with consumer-ready foods, such as the preparations of cereals. [106,107,108,109,110,111,112]. Up to a certain level, this study is similar to those dedicated to intra-industry competitiveness type of analyses [113,114,115], but particularly niched on the cereals sector.

**Table 1:** Description of the attributes and indicators included in the construction of the sustainable economic competitiveness index.

No.	Name of Attributes and Indicators	Reasoning	Data Source
Attribute 1: Factor Endowments and Resource Independence			
I_1.1_	The gross production value of cereals	In line with Porter’s Diamond model, the measurement of competitiveness needs to be approached through the lens of factor endowments, not only through processing capabilities or other influencing factors of competitiveness. Consequently, it is assumed in this study that countries with no cereal production capabilities cannot reach high levels of sustainable economic competitiveness. Not only that, but food security issues would eventually emerge, which would attract socioeconomic issues. Relying on a behavior characterized by importing cereal production from other countries does not converge with the concept of sustainable economic competitiveness. In such a context, price volatility would eventually negatively affect economic performance and food accessibility levels [90,116,117,118,119]. With the purpose of encapsulating the resource economics (I_1.1_) and resource management (I_1.2_) facets as two complementary determinants of sustainable economic competitiveness, both indicators were included in this *SECI* attribute.	FAOSTAT
I_1.2_	Cereal land allocation (the ratio between the cereal-harvested area and the total arable land)		FAOSTAT
Attribute 2: Agricultural Chain Performance			
I_2.1_	Cereal yield productivity expressed in tonnes of cereals per cereal-cultivated hectares	Yield productivity is a measurement of efficiency and economic performance that contributes to achieving higher levels of competitiveness [120,121,122]. This attribute of economic competitiveness provides robustness to the sustainability aspect of the constructed index.	FAOSTAT
I_2.2_	Cereal yield productivity expressed in thou-sand USD per cereal-cultivated hectares		FAOSTAT
Attribute 3: National Agricultural Chain Strategies and Policies			
I_3.1_	The ratio between the imports of cereals and the imports of the preparations of cereals	The literature converges towards the fact that the preparations of cereals deliver more competitiveness than the raw materials—cereals. This is grounded in the fact that exporting processed agri-food products implies many activities carried out to add more value to the final commercialized product [108,123,124,125]. By taking this value chain assessment framework into consideration, relevant indicators were included in the index composition. In addition, the design of the *SECI* respects the effects of national strategies and policies specific to the cereal chain: (a) the imports of cereals must exceed those of the preparations of cereals (I_3.1_); and (b) the exports of the preparations of cereals must exceed those of the cereals (I_3.2_). Since factor endowments and resource independence were already considered in the development of this index, the strategic processing flows and value chain management systems were taken into account by integrating indicators I_3.1_ and I_3.2_ into the composition of the *SECI*.	INTRACEN Trade Map
I_3.2_	The ratio between the exports of the preparations of cereals and the exports of cereals		INTRACEN Trade Map

Higher ratio values express high levels of agricultural chain competitiveness due to efficient national strategies and policies.

Cereals refer to crops harvested for dry grain only, in accordance with FAOSTAT’s methodology for crops and livestock [126]. It excludes cereal crops harvested for hay, food, feed, silage or used for grazing. FAOSTAT was the source of data related to the value of gross cereal production, which was compiled by multiplying the gross production in physical terms by output prices at farm gate. Regarding the other data source, INTRACEN Trade Map, cereals consist of many subgroups with their associated codes: 1001—wheat ad meslin; 1002—rye; 1003—barley; 1004—oats; 1005—maize and corn; 1006—rice; 1007—grain sorghum; 1008—buckwheat, millet, canary seed and other cereals. Data were extracted from FAOSTAT and INTRACEN Trade Map in September 2022 for the 2011–2020 period [127].

To provide more details regarding the sample of this study, data were gathered and computed with respect to the EU-27 member states, but with the exception of Cyprus, Luxembourg and Malta. One of the reasons for this decision is that those three countries generate together, on average, 0.10% of EU-27’s cereal production [128]. Therefore, it becomes impossible to describe those countries as potential leaders in terms of building sustainable economic competitiveness, as far as the cereals chain is concerned.

Data were normalized according to min–max normalization rescaling method, as described in Equation (1), where *x* represents the original value and *x′* represents the normalized value. The calibration of the attributes’ values was carried out based on a scale from zero to one. Zero was considered the least favorable outcome, while one was considered the most favorable—it describes the most competitive result. The rescaling was performed systematically, for each year included in the study, allowing the development of a comparison between each EU country within the sample, in terms of competitiveness, at the level of all index components.

(1) $$x^{\prime }=\frac{x-\min\left(x\right)}{\max\left(x\right)-\min\left(x\right)}$$

The mean, standard deviation and coefficient of variation were calculated for each of the indicators that compose the *SECI* for each country based on the data corresponding to the 2011–2020 period. This was performed with the purpose of checking data reliability and consistency. Following this, after having all the indicators from Table 1 rescaled, *SECI* was calculated according to Equation (2), where $\bar{I_{1.1}}$, $\bar{I_{1.2}}$, …, $\bar{I_{3.2}}$ represent the average value (*n* = 10 years) scored by the normalized indicators. *SECI* was calculated for each country.

(2) $$SECI=\frac{\bar{I_{1.1}}+\bar{I_{1.2}}+\bar{I_{2.1}}+\bar{I_{2.2}}+\bar{I_{3.1}}+\bar{I_{3.2}}}{6}$$

With the same scaling framework, *SECI* was normalized as described in Equation (3). Similarly, *SECI* represents the original value and **SECI*′* represents the normalized value. Scaled values that are closer to one describe high levels of competitiveness based on the attributes and components of *SECI*. The opposite was considered for values closer to zero. The construction of **SECI*′* and the research flow are presented in Figure 1.

(3) $$SECI^{\prime }=\frac{SECI-min\left(SECI\right)}{\max\left(SECI\right)-min\left(SECI\right)}$$

After computing the normalized *SECI* for each country and product, we resorted to the Balassa index with the aim of providing a more comprehensive approach of the sustainability of economic competitiveness. The most commonly used technique to quantify the level of international agri-food trade competitiveness is the Balassa index [18], as well as its derived versions, which has been widely used in practice [106,129,130,131]. This measuring technique has its limitations—some scholars argue that the Balassa index reflects export specialization with asymmetry issues [132], sometimes due to the different effects of agricultural policies [133]. Costinot et al., emphasized the fact that the Balassa index does not encapsulate the influencing factors that contribute to the generation of competitiveness [134]. However, most practitioners accept those limitations and enhance their research with other measurement techniques of competitiveness from its different facets [135,136,137]. The Balassa index, also referred to as the revealed comparative advantage (RCA), was studied in this empirical research as a complementary, yet insufficient method of the measurement of the sustainable economic competitiveness. This twofold approach allows a better understanding of capitalizing on *SECI*. As described in Equation (4), *RCA* expresses the ratio between the export market share of a country, a product, or a group of products and its export market share in the total trade in a set of countries.

(4) $$RCA_{ij}=\frac{X_{ij}}{X_{ik}}/\frac{X_{nj}}{X_{nk}}$$

In Equation (4), *X* represents the export value, *i* represents the country of analysis, *n* represents the whole EU, *j* represents the analyzed group of agri-products (either cereals or preparations of cereals, systematically approached, in accordance with the research objective), and *k* represents all agri-food traded goods (categories 1–24, as classified in the INTRACEN Trade Map database, which is the data source) [127]. Similarly, data were extracted for the 2011–2020 period. In accordance with the literature: *RCA* values below one justify no comparative advantage; *RCA* values positioned between one and two signal a weak comparative advantage; medium comparative advantage can be noticed if *RCA* values are positioned between two and four; and strong comparative advantage is expressed if *RCA* values exceed four. Thus, through the lens of the *RCA* index and normalized *SECI*, this paper provides a holistic approach and multiple perspectives on the sustainability of the economic competitiveness. *SECI* can represent a real solution for going beyond some of the Balassa index’s limitations [138], a complementary assessment method for sustainable economic competitiveness in the case of agri-food value chains.

The histogram of the normalized *SECI* was constructed, and the descriptive statistics were discussed. In addition, results were approached per country by index attributes: (a) factor endowments, resource independence; (b) agricultural chain performance; and (c) national agricultural chain strategies and policies. Following this, the correlation matrix was designed for the normalized *SECI* in relation with its six components and *RCA* for cereals and preparations of cereals.

Finally, multiple cross-sectional linear regression models were constructed using the least squares method with the aim of analyzing the impact of each *SECI* component on fostering sustainable economic competitiveness. All the econometric models consisted of two variables: (1) the normalized *SECI* acting as the dependent variable; and (2) systematically, each of the *SECI* components acting as predictors, from I_1.1_ to I_3.2_, as described in Figure 1. In this empirical study, the confidence level was 95%. Each regression model had 23 degrees of freedom. Equation (5) shows the regression equation:

(5) $$SECI^{\prime }=Constant\;coefficient+Coefficient\;of\;the\;predictor\times Predictor\;value+\varepsilon$$

By carrying out the empirical research in this manner, the influence of each index component on its normalized form was computed, therefore revealing the impact on the generation of sustainable economic competitiveness. In addition, to ensure a more comprehensive approach for this study, the *RCA* index was also considered when building the econometric models, as shown in Figure 1. In the case of both type of products analyzed, the *RCA* index acted as predictor for the normalized *SECI*, therefore revealing the relation between two different proxies aimed to quantify economic competitiveness.

## 3. Results and Discussion

In accordance with the methodology presented in Section 2, the first step was to study the descriptive statistics of the normalized indicators used to construct *SECI* based on a ten-year period: 2011–2020. Hence, Table 2 was initially designed for presenting the descriptive statistics of the raw data used in the *SECI* composition. Afterwards, the mean, standard deviation and coefficient of variation of the *SECI* normalized components were calculated in Table 3, per country and index component.

Considering the results regarding the mean, standard deviation and coefficient of variation of the index attributes from Table 3, the relative steadiness of the data shows the overall slow transition to patterns specific to sustainable economic competitiveness in the case of most EU member states. On average, the coefficient of variation registered values from 7.98% (minimum, in the case of I_1.2_) to 42.75% (maximum, in the case of I_2.2_). On the one hand, this proves that cereal land allocation (I_1.2_) did not show major signs of change in the EU. Even Portugal, the country that registered the maximum of 24.73%, shows signs of partial steadiness, because the ratio between the cereal-harvested area and the total arable land registered an average annual decrease of only 0.25 percentage points. On the other hand, in terms of cereal yield productivity expressed in thousand USD per cultivated hectares (I_2.2_), EU countries with poor performance in this regard also registered the greatest coefficient of variation: Estonia, Finland, Poland, Romania and Spain. In fact, those are the worst performing countries regarding this index component (I_2.2_), considering that the EU-27 average is 1.5 times bigger than the average of those five countries. Thus, the results hint at poor resource management systems, since the event of the standard deviation being so close to the mean occurs especially for the countries that are EU-27’s worst performers in terms of cereal yield productivity. In this regard, another form of consistency occurs—that specific to the inability of maintaining high cereal yield productivity if approached nationally, but low if approached at EU level. For example, the country that encounters the greatest issues in terms of maintaining or improving its results in terms of cereal yield productivity is Romania, a country that in 2020 registered half the productivity scored in 2011. The literature confirms poor resource management systems due to issues regarding inefficient land concentration [139], lack of cooperative ventures [140] and lack of performant agricultural technologies [141]—all converging towards the conclusion that investments are needed for the basic infrastructure in rural areas [142].

In terms of factor endowments and resource independence, France is the leader in terms of this *SECI* attribute, scoring the maximum values for both indicators that reveal this facet of agricultural competitiveness. France produces the most cereals among all the EU countries. These findings are validated by the standard deviation and coefficient of variation, which are zero, suggesting consistency throughout the analyzed period. In addition to this favorable position for France, its RCA for cereals is situated above the average, yet a trade-off was observed in relation with the RCA corresponding to the preparations of cereals, average RCA 0.94, signaling no comparative advantage in this regard, hence a lack of sustainable economic competitiveness. Although the performance of the French cereal chain is well above the average (see the components of attribute two from Table 3), France fails to capitalize on exporting more processed cereals. Instead, the export levels of raw cereals highly exceed those of exporting preparations of cereals, as tracked by *SECI* attribute three.

Germany finds itself in a similar position. The German cereal chain performance is slightly better than France’s, but Germany has a smaller production in comparison with France. Yet, they represent EU’s main players in terms of cereals production, followed by Poland, Romania, Spain, Italy and Hungary. Unlike France, Germany managed to score more favorable ratios: (a) between the imports of cereals and the imports of the preparations of cereals; and (b) between the exports of the preparations of cereals and the exports of cereals, which could be the result of a more developed agricultural infrastructure and processing capabilities. However, Germany does not register any comparative advantage in terms of the exports with cereals, which, on the other hand, is the case with France.

Italy has one of the most versatile strategies in relation to the cereal chain. While it registered the greatest comparative advantage in terms of exporting processed cereals, Italy does not possess as many raw materials as France, Germany or Poland. This can be noticed in the results from attribute one in Table 3. In addition, no comparative advantage was observed as far as exporting cereals was concerned. Not only that, but Italy was also in the leaderboard’s bottom tier in this regard. However, yield productivity delivered competitiveness for Italy. Moreover, the ratios encompassed in the *SECI* attribute three characterize a sustainable economic competitiveness of the Italian cereal chain. Italy is focused on importing cheap raw materials (cereals), processing it, adding value through a performance chain management system and exporting agri-food products with high levels of added value. This flow ensures its sustainable economic competitiveness, but attention should be paid to the domestic cereal production and price volatility.

Up to a certain level, the Netherlands follows Italy’s pattern, but it is deficient in terms of the production of cereals. The results concerning the ratio between the Dutch exports of the preparations of cereals and the exports of cereals signal one of the most competitive national agricultural chain strategies among all the EU countries. However, the country is highly reliant on importing cereals due to the low volumes of cereal production. In addition, Portugal’s *SECI* follows the same patterns as the Netherlands’ but at a smaller scale, and it is negatively influenced by the yield productivity results. The same productivity issues were observed in the case of its neighbor, Spain.

Ireland, the Netherlands and Belgium are the most competitive EU countries in terms of cereal chain performance, registering the greatest cereal yield productivity, expressed both in terms of tonnes of cereals per cultivated hectares and thousand USD per cereal-cultivated hectares. However, those three countries lack factor endowments, especially in terms of the value of the cereal production. Although Ireland and Belgium have paid attention to land allocation, this is not enough to compensate the poor performance results regarding cereal production. However, what adds more value to their sustainable economic competitiveness is their efficient national chain strategies and policies that focus on obtaining highly favorable ratio results concerning the ratio between the exports of the preparations of cereals and the exports of cereals. Regarding this *SECI* index attribute, the results of Ireland place the country so far ahead of the other EU countries that additional attention was paid during the normalization process for this particular index component.

**Figure 2:**
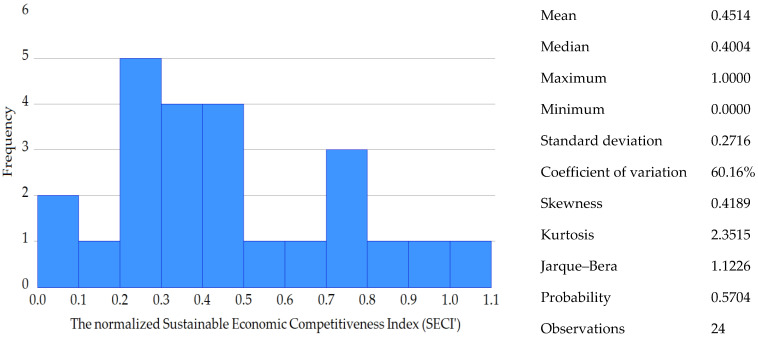
The histogram of the normalized sustainable economic competitiveness index (*SECI*′).

**Figure 3:**
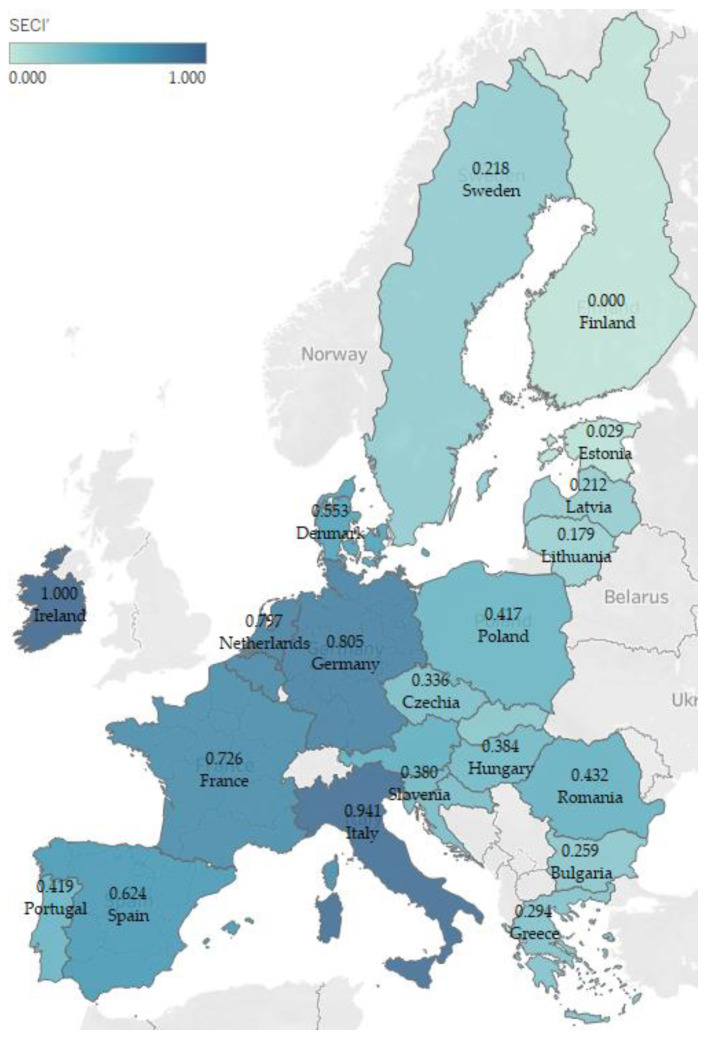
The normalized sustainable economic competitiveness index (*SECI*′) per country.

**Figure 4:**
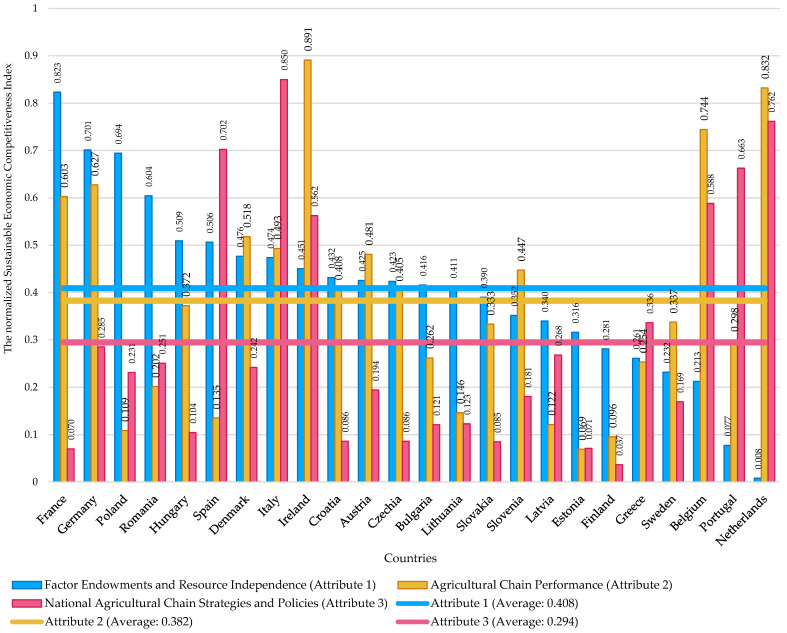
The sustainable economic competitiveness index attribute results per country.

Research findings stand proof of a competitiveness trade-off in the case of the cereal chain from Ireland, the Netherlands, Belgium and Portugal. It is a resource management paradox caused by many factors: a trade balance deficit that occurs for cereals, simultaneous with scoring the best comparative advantage in terms of the preparations of cereals. The opposite was observed for countries such as Romania, Bulgaria and Hungary. In the case of those countries, they have relatively high cereal production volumes; they are the most competitive in terms of exporting raw cereals—the production of cereals allows scoring considerable trade balance surplus levels in this regard; but they cannot capitalize on the processing capabilities. This negatively affects their value chain system and triggers poor levels of sustainable economic competitiveness, despite the abundance of natural factor endowments. While Figure 2 was dedicated to the histogram of the normalized *SECI*, the results per country were displayed in Figure 3, and Figure 4 was designed to show the results per index attribute.

At the level of the analyzed EU countries, the mean of normalized *SECI* is 0.4514. Its distribution is platykurtic and positively skewed. This type of distribution confirms that most of the observations do not have high levels of sustainable economic competitiveness in the case of cereals chain and that there are very few outliers that fall in favorable positions in this regard: Ireland, Italy, Germany, the Netherlands, Belgium and France. However, at a more in-depth analysis at the level of index components, each of those leaders have some weaknesses. As previously explained: (a) Ireland and Belgium lack cereal production capabilities when compared to the EU average; (b) Italy heavily relies on the imports of cereals; (c) France performs poorly at exporting preparations of cereals; (d) Germany’s situation is similar to France’s, but the production quantity of cereals is not as big in volume as France’s, and the German exporting and importing ratios are more favorable than France’s; (e) the Netherlands’ cereal import dependence represents a major weakness for their sustainable economic competitiveness, but in comparison with Italy, the Dutch yield productivity is much better.

At the opposite side of sustainable economic competitiveness, there are countries such as Estonia, Finland and Lithuania, situated at the bottom leaderboard of *SECI*. This is due to various causes: (a) in comparison with the rest of the analyzed countries, their production quantity of cereals is insignificant; (b) yield productivity is poor; (c) they lack a solid sustainable strategy for the trade flows with cereals and derived products. Results from Table 3 confirm those findings, despite the fact that those countries have medium comparative advantages in terms of cereal exports.

As far as Spain is concerned, the country plays a significant part in EU’s cereal trading dynamics. In 2020, Spain produced 27,320,900 tonnes of cereals—almost 10% of EU-27’s total cereal production. However, in the same year, it registered a cereal trade balance deficit of USD 2,495,581 thousand and surplus of USD 644,884 thousand in the case of the preparations of cereals. No comparative advantages were observed in either direction. The yield productivity results signal possible deficiencies in the Spanish agricultural infrastructure. Based on the *SECI* composition, Spain excels at importing more cereals than processed cereals. Following this trade pattern, Spain harnesses competitiveness by exporting high levels of processed cereals, instead of exporting raw materials, which it possesses regardless. However, the processing infrastructure needs to be improved to become more sustainable economic competitive.

Following this, the correlation matrix was elaborated in Table 4. This is an initial step before constructing the cross-sectional linear regression models that allow the study of the impact of the index components and of the RCAs on the generation of sustainable economic competitiveness in the European cereal chain.

The normalized *SECI* is highly and positively correlated (0.8546) with cereal yield productivity expressed in thousand USD per cultivated hectares, immediately followed by the ratio between the exports of the preparations of cereals and the exports of cereals (0.7564). This ratio is moderately and negatively correlated with the Balassa index for raw materials, cereals (−0.6481), which follows one of the main ideas of this article—that sustainable economic competitiveness requires capitalizing comparative advantages at exporting high-added-value products instead of raw materials (cereals) with little added value. In addition, almost all correlation results with respect to the Balassa index for cereals are negatively correlated with the rest of the *SECI* components. These empirical research findings confirm comparative advantage trade-offs between agri-food products traded during different processing stages. Additionally, the same findings from the correlation matrix emphasize the previously explained resource management paradox caused by the lack of performant agricultural infrastructure and poor national foreign trade strategies and policies. To better support this finding, another argument resides in the opposite directions of poor foreign trade strategies (focused on increasing the Balassa index for cereals) and highly competitive foreign trade strategies (focused on increasing the Balassa index for preparations of cereals), which is expressed by their corresponding negative correlation result: −0.4612.

Finally, eight cross-sectional linear regression models were constructed in Table 5 with the aim of providing robustness to the research findings and determining the impact of each index component on delivering sustainable economic competitiveness in the EU. RCAs for cereals and preparations of cereals were also considered, in accordance with the research methodology presented in Section 2.

According to the results from Table 5, the most impactful component was I_2.2_. Its corresponding coefficient of determination confirms that 73.04% of the variation of the normalized *SECI* is explained by the variation of the cereal yield productivity expressed in thousand USD per cereal-cultivated hectares. The corresponding *p*-value of the constant coefficient (0.0761) is slightly above the 0.05 threshold, but it is accepted, and the model is considered valid. To continue the analysis of index component impact on the generation of sustainable economic competitiveness, results confirm that the second most influential component was I_3.2_. Its corresponding coefficient of determination confirms that 57.21% of the variation of the normalized *SECI* is explained by the variation of the ratio between the exports of the preparations of cereals and those of raw cereals. The *p*-values of those coefficients are below the 0.05 accepted threshold. Surprisingly, the variation of RCAs cannot explain the variation of the normalized *SECI*, although the RCA corresponding to the preparations of cereals is worth mentioning due to the level of 38.30% variation explanation. These econometric research findings emphasize that the most efficient way to deliver sustainable economic competitiveness is ensuring high levels of yield productivity, simultaneously with following strategic foreign trade patterns: export more processed agri-food products with high added value than exporting raw materials with little added value. Tracking value chain flows is essential for ensuring high levels of sustainable economic competitiveness.

The findings of this empirical research paper are supported by the body of literature dedicated to the topic of agri-food value chain management with a particular focus on the strategic actions for value creation along the chain links, hence contributing to building sustainable economic competitiveness [3,34,76,77,78,79,80,82,143,144,145,146,147,148,149]. To provide more literature evidence that supports the main highlights of this empirical paper, findings are convergent towards the following:

- Competitiveness shortcomings exist especially in the dynamics of food processing capabilities and international food trade patterns [106,107];
- Productivity in the agri-food sector is a vector of competitiveness that should be empowered starting from the primary agriculture and should be harnessed even in food processing stages to ensure sustainable economic competitiveness [108];
- Agri-food products with low added value can significantly contribute to worsening the trade balance results and diminishing competitiveness levels [109];
- Raw agricultural materials can deliver strategic competitiveness if they are involved in processing activities and, later, as a result of beneficial trade flows, they manage to generate high levels of comparative advantages [110];
- Administrative measures, national strategies, production techniques and input prices are factors that can significantly influence competitiveness levels—in this regard, Rumankova et al. [111] provided the Netherlands and Belgium as positive examples. The same happened in this empirical research—those two countries are leaders from the perspective of the competitiveness drivers previously mentioned;
- Upgrading agri-food value chains at the economic level: product, process, functional and inter-sectoral is essential for capturing higher benefits and competitiveness [112];
- Despite being interconnected, agriculture and the food industry can follow divergent trends as far as building competitive performance is concerned [113,114,115].

This article complements the literature dedicated to agri-food competitiveness. Economic competitiveness and sustainability were acknowledged as two subjective and complex concepts, and, instead of aiming to measure only comparative advantages, this research brings its contribution with a niched tool designed to measure the level of sustainable economic competitiveness in the agri-food value chains–*SECI*.

## 4. Conclusions

Competitiveness and sustainability are two complex concepts that have been highly debated in the literature. There are many valences and nuances to those illusive concepts. Their meanings are different from stakeholder to stakeholder, decision-maker to decision-maker, scholar to scholar. On top of that, agricultural competitiveness adds more complexity to the sustainability of agricultural competitiveness, which is inevitably tied to foreign trade flows and to capitalizing on competitive advantages. It is generally accepted in the literature that competitive advantages can be turned into sustainable competitive advantages if favorable market positions are maintained over time, as well as by constantly improving the product through technological and strategic methods. Of course, the international political climate, governmental impacts and the constantly changing business environment are influencing factors to be taken into account when designing strategies for ensuring the sustainability of high levels of competitiveness.

This research paper proposed a niched approach to the sustainability of agricultural competitiveness with a particular focus on the economic dimension. By consulting relevant papers in the field, the sustainable economic competitiveness index (*SECI*) was constructed, aiming to have direct application for agri-food value chains. This index consists of three attributes: (a) factor endowments and resource independence; (b) agricultural chain performance; (c) national agricultural chain strategies and policies. The cereal chain was the subject of analysis for a selection of EU countries. Findings show that building agricultural sustainable economic competitiveness relies on finding an ‘equilibrium’ between the management of national factor endowments and the efficient management of international trade flows. Adding more layers to the managerial implications of this paper, those not only come in the support of decision-makers. Two different types of agricultural competitiveness trade-offs were explained in this paper, requiring the attention of scholars and practitioners. The Netherlands, Ireland, Belgium and Portugal face a resource management paradox characterized by a trade balance deficit for cereals, while scoring high levels of competitiveness in terms of exporting preparations of cereals—this is not necessarily economically sustainable because of cereal price volatility and resource dependence. On the other hand, there are countries such as Romania, Bulgaria and Hungary with relatively high cereal production volumes at the level of the EU; they are highly competitive in terms of exporting cereals, allowed by their cereal production capabilities. However, those countries cannot capitalize on cereal processing, which negatively impacts their sustainable economic competitiveness level, despite their abundance of resource endowments. Thus, one of the key vectors for building sustainable economic competitiveness is delivering smart, not more—the focus should be on exporting agri-food products with high levels of added value, instead of exporting huge volumes of raw agricultural materials that have little added value. As noticed, those materials usually return through import flows under the form of processed foods but at a higher price.

As far as the CAP is concerned, building the competitiveness of SMEs is one of the most well-financed directions from the European agricultural fund for rural development and national budgets during the 2014–2020 programming period: EUR 43.391 billion [150]. This approach is synergic with building sustainable economic competitiveness, since all the links involved in the agri-food value chain should strive to reach their potential and focus around capitalizing on their competitive advantage. Thus, a similar approach is recommended for the 2021–2027 programming period for the objective of building competitiveness. Moreover, the results of this empirical study come to support the CAP with the following policy recommendations: taking advantage of national factor endowments as a vector of resource independence is highly valuable for maintaining the sustainability of economic competitiveness. Taking the EU’s objective of cohesion into consideration [151,152], investments are still needed in the agricultural infrastructure, especially in countries abundant in resources such as agricultural land and raw materials, but that lack processing capabilities and proper market access.

The authors acknowledge that this research has some limitations: (a) the constructed index has only three attributes with two components each—yet more are needed to fully capture the image of the sustainable economic competitiveness level—this can be further developed in future studies; (b) although *SECI* has direct application in the case of agri-food chains and can be easily applied for other agri-food chain besides the one analyzed in this study, the cereal chain, it needs to be further refined to properly function for other economic sectors; (c) the normalized *SECI* values can suggest that certain countries are absolute winners in terms of building sustainable economic competitiveness. Yet, this is not true—values of one or closer to one were attributed only in relation with the rest of sample observations in the rescaling process. In this regard, improving competitiveness levels needs to be harmonized per index attribute and per component (see Figure 4).

As a future research direction concerning agricultural competitiveness, it would be worth studying the paradox of conflicting objectives. It occurs when, on the one hand, (1) each country strives to improve its economic performance in terms of exporting agri-food products more efficiently by relying on price or volume strategies, and, on the other hand, (2) the 2030 Agenda for Sustainable Development calls for achieving high food security levels through SDG-2: Zero Hunger. As a consequence, striving to become more competitive through increasing production capabilities—only later to export more agri-food products to countries that offer the best price—this might actually trigger negative unwanted effects in relation with the common goal of ensuring nutritious food access to all people, particularly to those in vulnerable situations, at all times, as described in the first target of SDG-2. Finding pathways for achieving both objectives is still challenging. Thus, future research avenues could tap into the design of an index that would encompass both the sustainable economic competitiveness components, as well as the food security components.

## Figures and Tables

**Table 2:** Descriptive statistics of the indicators used to construct the sustainable economic competitiveness index (*SECI*).

Indicator	I_1.1_	I_1.2_	I_2.1_	I_2.2_	I_3.1_	I_3.2_
Mean	2,440,905.325	0.509	5.498	1.112	0.788	5.258
Median	980,151.850	0.541	5.418	1.037	0.561	1.467
Maximum	13,289,127.900	0.696	8.760	2.108	2.287	77.933
Minimum	112,291.100	0.180	3.532	0.641	0.099	0.073
Standard deviation	3,237,247.225	0.119	1.570	0.359	0.606	15.633
Skewness	2.061	−1.197	0.516	0.997	1.102	4.445
Kurtosis	6.807	4.261	2.273	3.806	3.259	21.192
Jarque−Bera	31.477	7.326	1.593	4.627	4.928	409.981
Probability	0.000	0.026	0.451	0.099	0.085	0.000
Observations	24	24	24	24	24	24

**Table 3:** Descriptive statistics of the normalized indicators used to construct the sustainable economic competitiveness index (*n* = 10 years; 2011–2020).

Attribute	Attribute 1—Factor Endowments and Resource Independence						Attribute 2—Agricultural Chain Performance						Attribute 3—National Agricultural Chain Strategies and Policies					
Indicator	I_1.1_			I_1.2_			I_2.1_			I_2.2_			I_3.1_			I_3.2_		
Country	Mean	Standard Deviation	Coefficient of Variation	Mean	Standard Deviation	Coefficient of Variation	Mean	Standard Deviation	Coefficient of Variation	Mean	Standard Deviation	Coefficient of Variation	Mean	Standard Deviation	Coefficient of Variation	Mean	Standard Deviation	Coefficient of Variation
Austria	0.059	0.008	13.43%	0.791	0.020	2.53%	0.624	0.126	20.25%	0.337	0.128	37.81%	0.173	0.043	24.64%	0.215	0.066	30.78%
Belgium	0.025	0.003	11.79%	0.400	0.039	9.63%	0.978	0.070	7.20%	0.511	0.174	33.98%	0.493	0.050	10.19%	0.683	0.120	17.53%
Bulgaria	0.126	0.022	17.63%	0.706	0.050	7.07%	0.315	0.123	38.92%	0.209	0.052	25.09%	0.224	0.061	26.98%	0.018	0.005	27.81%
Croatia	0.033	0.007	19.71%	0.830	0.063	7.62%	0.536	0.177	33.01%	0.280	0.080	28.71%	0.075	0.019	26.00%	0.098	0.042	43.30%
Czechia	0.115	0.018	15.72%	0.732	0.029	3.93%	0.454	0.091	20.15%	0.355	0.123	34.72%	0.078	0.012	15.09%	0.094	0.028	29.66%
Denmark	0.136	0.013	9.88%	0.817	0.040	4.95%	0.583	0.083	14.22%	0.453	0.129	28.53%	0.139	0.026	18.85%	0.345	0.133	38.46%
Estonia	0.008	0.004	50.53%	0.624	0.059	9.43%	0.097	0.091	93.36%	0.041	0.052	126.79%	0.075	0.063	82.97%	0.067	0.026	38.51%
Finland	0.045	0.005	11.99%	0.517	0.067	12.89%	0.116	0.057	49.59%	0.075	0.056	73.97%	0.000	0.000	0.00%	0.073	0.028	38.40%
France	1.000	0.000	0.00%	0.647	0.027	4.20%	0.682	0.077	11.35%	0.523	0.180	34.44%	0.078	0.016	20.16%	0.062	0.014	22.96%
Germany	0.704	0.056	7.92%	0.698	0.020	2.85%	0.701	0.067	9.51%	0.554	0.175	31.51%	0.281	0.040	14.32%	0.289	0.105	36.41%
Greece	0.068	0.013	19.70%	0.455	0.085	18.68%	0.209	0.064	30.65%	0.298	0.096	32.31%	0.500	0.069	13.88%	0.172	0.062	35.90%
Hungary	0.199	0.036	17.84%	0.819	0.077	9.43%	0.465	0.170	36.57%	0.280	0.087	30.95%	0.198	0.044	22.38%	0.010	0.004	39.71%
Ireland	0.039	0.011	27.23%	0.862	0.061	7.10%	0.849	0.100	11.78%	0.934	0.138	14.82%	0.125	0.041	32.74%	1.000	0.000	0.00%
Italy	0.363	0.049	13.53%	0.584	0.047	8.11%	0.422	0.073	17.17%	0.564	0.170	30.17%	0.920	0.090	9.78%	0.780	0.142	18.25%
Latvia	0.029	0.011	37.05%	0.650	0.092	14.15%	0.143	0.090	62.85%	0.100	0.054	54.23%	0.517	0.167	32.35%	0.019	0.011	58.08%
Lithuania	0.068	0.017	24.35%	0.754	0.106	14.11%	0.162	0.079	48.41%	0.130	0.058	44.74%	0.219	0.078	35.88%	0.027	0.011	41.89%
Netherlands	0.016	0.002	13.88%	0.000	0.000	0.00%	0.917	0.058	6.30%	0.747	0.202	27.04%	0.591	0.113	19.19%	0.932	0.147	15.74%
Poland	0.388	0.062	16.04%	1.000	0.000	0.00%	0.158	0.076	47.93%	0.059	0.042	71.66%	0.216	0.054	25.22%	0.247	0.123	50.00%
Portugal	0.014	0.001	9.87%	0.141	0.035	24.73%	0.273	0.068	25.04%	0.324	0.065	19.91%	0.658	0.055	8.41%	0.668	0.244	36.55%
Romania	0.364	0.083	22.68%	0.845	0.046	5.43%	0.213	0.201	93.95%	0.190	0.134	70.55%	0.501	0.153	30.43%	0.000	0.000	0.00%
Slovakia	0.051	0.010	20.20%	0.729	0.034	4.63%	0.397	0.148	37.17%	0.269	0.099	36.95%	0.132	0.048	36.21%	0.038	0.008	21.70%
Slovenia	0.000	0.000	0.00%	0.703	0.034	4.88%	0.546	0.149	27.35%	0.348	0.136	39.16%	0.192	0.042	21.69%	0.170	0.055	32.32%
Spain	0.385	0.084	21.84%	0.628	0.040	6.33%	0.112	0.111	99.07%	0.158	0.104	65.48%	0.984	0.038	3.91%	0.420	0.075	17.78%
Sweden	0.067	0.009	12.79%	0.397	0.035	8.81%	0.411	0.111	27.10%	0.263	0.086	32.56%	0.065	0.018	26.81%	0.274	0.093	33.93%

**Table 4:** The correlation matrix of the normalized sustainable economic competitiveness index (*SECI*′).

Correlation Probability	*SECI*′	I_1.1_	I_1.2_	I_2.1_	I_2.2_	I_3.1_	I_3.2_	RCA_c_	RCA_pc_
*SECI*′ *p*-value	1.0000								
	N/A								
I_1.1_ *p*-value	0.4259	1.0000							
	0.0380	N/A							
I_1.2_ *p*-value	−0.0865	0.2297	1.0000						
	0.6876	0.2803	N/A						
I_2.1_ *p*-value	0.7110	0.0871	−0.1821	1.0000					
	0.0001	0.6858	0.3945	N/A					
I_2.2_ *p*-value	0.8546	0.1461	−0.2303	0.8612	1.0000				
	0.0000	0.4957	0.2790	0.0000	N/A				
I_3.1_ *p*-value	0.4234	0.0918	−0.4071	−0.1067	0.1060	1.0000			
	0.0392	0.6696	0.0484	0.6197	0.6220	N/A			
I_3.2_ *p*-value	0.7564	−0.1135	−0.4760	0.5557	0.7536	0.4738	1.0000		
	0.0000	0.5974	0.0187	0.0048	0.0000	0.0194	N/A		
RCA_c_ *p*-value	−0.4028	0.1082	0.3571	−0.3722	−0.4375	−0.1495	−0.6481	1.0000	
	0.0510	0.6147	0.0867	0.0733	0.0325	0.4858	0.0006	N/A	
RCA_pc_ *p*-value	0.6188	0.0526	0.1114	0.5173	0.6812	−0.0784	0.6409	−0.4612	1.0000
	0.0013	0.8072	0.6044	0.0096	0.0002	0.7158	0.0007	0.0233	N/A

RCA_c_ = The Balassa index for cereals; RCA_pc_ = The Balassa index for preparations of cereals; N/A = not applicable.

**Table 5:** The output of the cross-sectional linear regression models.

Cross-Sectional Linear Regression Output	Predictors of *SECI*′							
	I_1.1_	I_1.2_	I_2.1_	I_2.2_	I_3.1_	I_3.2_	RCA_c_	RCA_pc_
Coefficient of Determination (R^2^)	0.1814	0.0075	0.5056	0.7304	0.1793	0.5721	0.1623	0.3830
Adjusted R^2^	0.1442	−0.0376	0.4831	0.7181	0.1420	0.5527	0.1242	0.3549
Durbin–Watson	1.8453	1.8310	1.7690	2.4893	1.8566	1.9067	1.8460	2.2267
F–statistic	4.8747	0.1660	22.4946	59.5934	4.8066	29.4172	4.2612	13.6546
*p*-value (F–statistic)	0.0380	0.6876	0.0001	0.0000	0.0392	0.0000	0.0510	0.0013
Estimation equation: *SECI*′ = Constant coefficient + Coefficient of the predictor × Predictor value + ε								
Constant Coefficient	0.3674	0.5168	0.1362	0.1008	0.3210	0.2648	0.5562	0.1238
Standard Error of the Constant Coefficient	0.0639	0.1701	0.0775	0.0541	0.0786	0.0506	0.0726	0.0992
*p*-value (Constant Coefficient)	0.0000	0.0060	0.0929	0.0761	0.0005	0.0000	0.0000	0.2251
Coefficient of the Predictor	0.4688	−0.1024	0.7301	1.0515	0.4212	0.6686	−0.0586	0.3627
Standard Error of the Predictor Coefficient	0.2123	0.2512	0.1539	0.1362	0.1921	0.1233	0.0284	0.0982
*p*-value (Coefficient of the Predictor)	0.0380	0.6876	0.0001	0.0000	0.0392	0.0000	0.0510	0.0013

## Data Availability

The raw data used in this empirical research were taken from FAOSTAT, Food and Agriculture Organization of the United Nations (FAO—email: faostat@fao.org; contact phone number: 390657052548; website: https://www.fao.org/faostat/; accessed on 19 September 2022) and INTRACEN Trade Map (email: marketanalysis@intracen.org; contact phone number: 410227300540; website: https://www.trademap.org/; accessed on 19 September 2022).
